# Open Reduction and Internal Fixation of Simultaneous, Bilateral, Atypical Femoral Shaft Fractures After Nine Years of Bisphosphonate Treatment

**DOI:** 10.7759/cureus.19177

**Published:** 2021-11-01

**Authors:** Tatsuki Kobayashi, Tetsuhiro Ishikawa, Joe Katsuragi, Yasuhito Sasaki, Seiji Ohtori

**Affiliations:** 1 Orthopedic Surgery, Sanmu Medical Center, Chiba, JPN; 2 Orthopedic Surgery, Graduate School of Medicine, Chiba University, Chiba, JPN

**Keywords:** autobiographical case report, open reduction and internal fixation, bilateral, simultaneous, bisphosphonate, atypical fracture

## Abstract

Bisphosphonates are generally used to treat osteoporosis and decrease the risk of femoral neck and vertebral fractures in patients with osteoporosis. Recently, it has been suggested that long-term bisphosphonate use can lead to decreased bone remodeling and an increased risk of atypical fractures. Atypical fractures often occur in the femur. The purpose of the present report is to describe a rare case of simultaneous, bilateral, atypical femoral fractures. An 80-year-old female was walking when she sustained bilateral femoral fractures that were diagnosed as atypical. The patient had received bisphosphonate treatment over the prior nine years.

## Introduction

Bisphosphonates are one of the most prescribed drugs for the treatment of osteoporosis because they decrease the risk of femoral neck and vertebral fractures in patients with osteoporosis [1,2]. Bisphosphonate therapy can increase bone marrow density (BMD), although the BMD response typically reaches a plateau in two to three years [3].

There is evidence to suggest that prolonged use of bisphosphonates can suppress bone remodeling and increase the risk of atypical fractures. For this reason, physicians can consider a drug holiday after long-term bisphosphonate use (three to 10 years) to reduce the risk of such complications [1]. Reportedly, several prodromal symptoms are likely reported before atypical femoral fractures (AFFs) occur and physicians need to be aware of them in the patients for whom they are prescribing antiresorptive drugs [2].

Atypical fractures occur frequently in the femoral subtrochanteric region or shaft [2]. Most reports of AFFs refer to unilateral cases. Here we describe a rare case of simultaneous, bilateral AFFs.

## Case presentation

**Figure 1:**
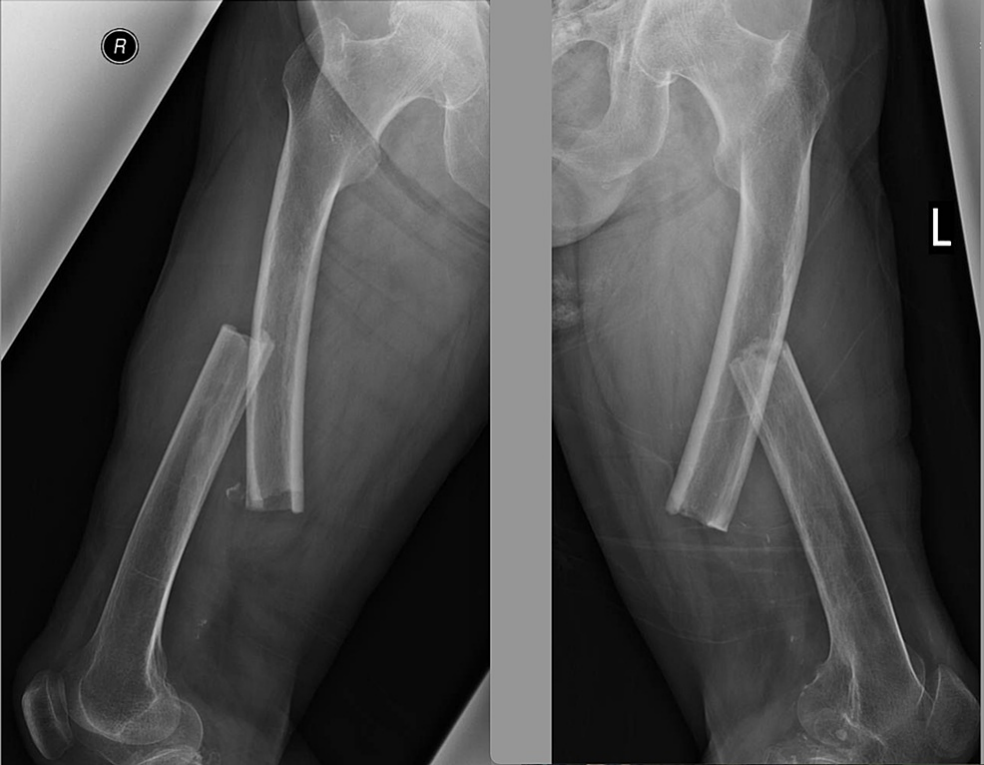
Plain radiographs of the right and left femurs. Bilateral transverse fractures with cortical thickening suggest the fractures were atypical.

An 80-year-old female fell when walking. At the moment of catastrophic injury, the patient described hearing a noise coming from both thighs before falling to the ground. A radiographic examination showed bilateral femoral shaft fractures (Figure 1). 

**Figure 2:**
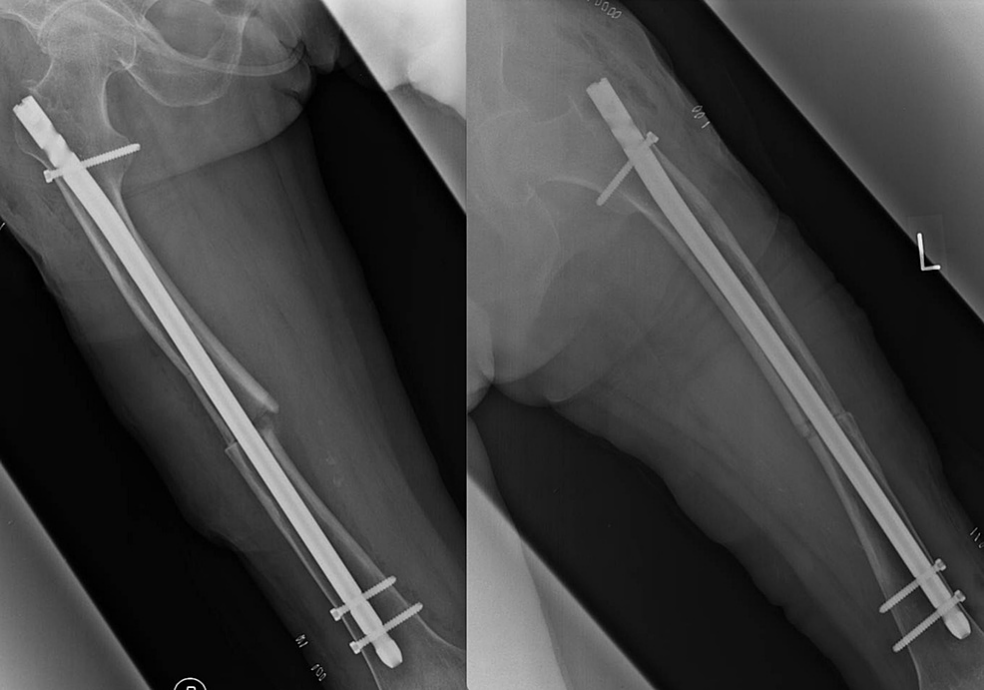
Plain radiographs of the right and left femurs immediately after surgery.

Her medical history revealed that she had been prescribed 2.5 mg/day of risedronate for the prior nine years as a treatment for osteoporosis. Radiographs and computed tomographic (CT) images revealed a transverse fracture in the middle of each femur, and cortical thickening was evident at both fracture sites. A dual-energy X-ray absorptiometry scan showed that her lumbar vertebral BMD was 54% of that of adults aged 20-44 years. The patient's T-score in the spine was -3.7 and Z-score was -0.1. Blood tests indicated that her serum intact amino-terminal propeptide of type 1 procollagen was 128 μg/L (26.4-98.2) and her tartrate-resistant acid phosphatase 5b was 541 μU/dL (120-420). The patient was diagnosed with simultaneous bilateral AFFs. Open reduction and internal fixation of the fractures were performed using an intramedullary (IM) nail system (T2 Nail, Stryker Corp., Kalamazoo, MI) (Figure 2). 

**Figure 3:**
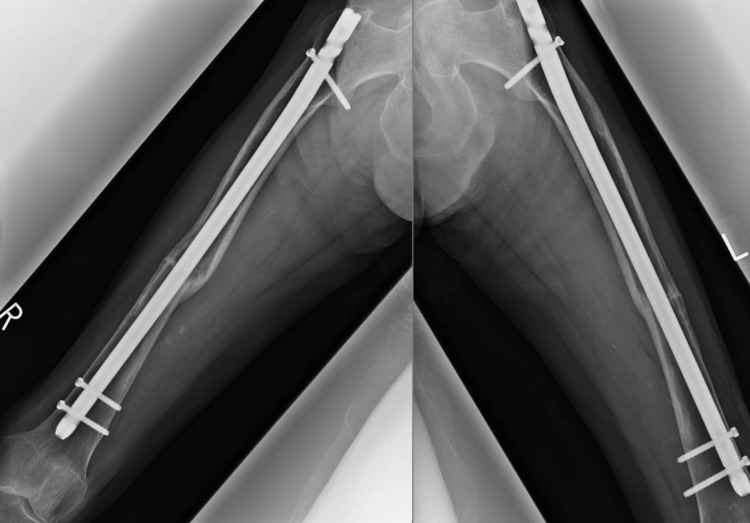
Plain radiographs of the right and left femurs one year after surgery when bone union was observed.

It was imperative for the patient to not bear weight on either leg for six weeks after surgery. During the four months of hospitalization, walking practice was included as part of the patient’s rehabilitation until she was eventually discharged with a walking cane. Bone union was observed and eight months after her surgeries, she was able to walk without the assistance of a cane (Figure 3).

## Discussion

The American Society of Bone and Mineral Research Task Force case definition of AFFs is shown in Table 1 [1]. This case report exhibited features similar to those typically observed as major features of AFFs, such as no associated trauma or minimal trauma after falling from a standing height or less. As shown in Table 1, other examples of major features are a transverse or short oblique configuration, noncomminuted, complete fractures extending through both cortices which may be associated with a medial spike, or incomplete fractures only involving the lateral cortex. Minor features of AFFs were also evident in this case; there was a generalized increase in shaft cortical thickness, bilateral fractures, and bilateral prodromal pain at the thighs.

**Table 1:** The ASBMR Task Force case definition of AFFs. AFF: atypical femur fracture; ASBMR: American Society for Bone and Mineral Research; BP: bisphosphonate ^a^All major features are required to satisfy the case deﬁnition of AFF. None of the minor features are required but have been sometimes associated with these fractures. ^b^Often referred to in the literature as beaking or ﬂaring.

Major features^a^
Located anywhere along the femur from just distal to the lesser trochanter to just proximal to the supracondylar flare
Associated with no trauma or minimal trauma, as in a fall from a standing height or less
Transverse or short oblique configuration
Noncomminuted
Complete fractures extend through both cortices and may be associated with a medial spike; incomplete fractures involve only the lateral cortex
Minor features
Localized periosteal reaction of the lateral cortex^b^
Generalized increase in cortical thickness of the diaphysis
Prodromal symptoms such as dull or aching pain in the groin or thigh
Bilateral fractures and symptoms
Delayed healing
Comorbid conditions (e.g., vitamin D deficiency, rheumatoid arthritis, hypophosphatasia)
Use of pharmaceutical agents (e.g., BPs, glucocorticoids, proton pump inhibitors)
Speciﬁcally excluded are fractures of the femoral neck, intertrochanteric fractures with spiral subtrochanteric extension, pathological fractures associated with primary or metastatic bone tumors, and periprosthetic fractures.

Bisphosphonates are among the most prescribed drugs for the treatment of osteoporosis to reduce the risk of bone fractures. Bisphosphonates increase BMD, although the BMD response typically reaches a plateau in two to three years [3]. In contrast, in a systematic review of all case reports and case series of AFFs, Park-Wyllie et al. reported a median time of five years of bisphosphonate therapy prior to fracture [4]. AFFs represent approximately 0.5-1% of proximal femoral fractures [2]. There are few case reports of simultaneous bilateral AFFs; only seven published reports were retrieved during our search (Table 2). The duration of bisphosphonate use was only reported in five of seven published cases [5-10]. Three of those five documented cases were reports of bisphosphonate use for more than five years (Table 2) [5-7]. In our case, the patient discontinued her bisphosphonate regimen and received (SR3 and SR4) 1 μg/day of alfacalcidol and 800 mg/day of calcium L-aspartate hydrate.

**Table 2:** Past reports of cases of simultaneous bilateral AFF. AFF = atypical femur fracture

Reports of cases	Reference number	Age/sex	Bisphosphonate	Duration ( years)	Mechanism of injury	Prodromal symptoms
Kamijo et al.	[5]	59/F	Alendronate	4	Fall	(-)
		74/F	Minodronic acid	7	Fall	(+)
Higgins et al.	[6]	71/F	Alendronate	8	Fall	(+)
Zafeiris et al.	[7]	76/M	Alendronate	11	Fall	(+)
Puah and Tan	[8]	64/M	Alendronate	1	Fall	(+)
Ninomiya et al.	[9]	80/F	-	-	-	-
Ovaska et al.	[10]	60/F	Risedronate	-	-	-

For several weeks or months before an AFF occurs, approximately 70% of patients present with groin or thigh pain [1]. For patients who have been prescribed antiresorptive drugs (e.g., bisphosphonates or denosumab) and report such symptoms, physicians should consider performing full-length radiographs of both femurs [11]. Githens et al. suggested that if the patient is asymptomatic but presents with radiographic signs that raise concern about an impending atypical fracture, prophylactic IM nailing is recommended [12]. It has been shown that administration for teriparatide treatment in patients with an atypical fracture may help facilitate fracture healing, recovery of hip function, and pain relief when bone turnover is reduced [13].

## Conclusions

In summary, this case report describes AFFs that occurred bilaterally and concurrently. The fractures were associated with long-term bisphosphonate use. Open reduction and internal fixation of the fractures were performed using an IM nail system. Bone union was achieved, and the patient was eventually able to walk again without assistance. Typically, AFFs occur unilaterally, although in rare cases, they can occur bilaterally. Physicians need to be aware of the effects of prescribing bisphosphonates for prolonged periods and need to consider a drug holiday after long-term bisphosphonate therapy to reduce the risk of atypical fractures. Another important point is to not overlook the prodromal symptoms of patients.
